# Baru Pulp (*Dipteryx alata* Vogel): Fruit from the Brazilian Savanna Protects against Oxidative Stress and Increases the Life Expectancy of *Caenorhabditis elegans* via SOD-3 and DAF-16

**DOI:** 10.3390/biom10081106

**Published:** 2020-07-25

**Authors:** Natasha Rios Leite, Laura Costa Alves de Araújo, Paola dos Santos da Rocha, Danielle Araujo Agarrayua, Daiana Silva Ávila, Carlos Alexandre Carollo, Denise Brentan Silva, Leticia Miranda Estevinho, Kely de Picoli Souza, Edson Lucas dos Santos

**Affiliations:** 1 Research group on Biotechnology and Bioprospecting applied to metabolism (GEBBAM), Federal University of Grande Dourados, Rodovia Dourados-Itahum, Km 12, Dourados 79804-970, MS, Brazil; natasha_riosleite@hotmail.com (N.R.L.); laurazootecnia@gmail.com (L.C.A.d.A.); paolarocha.biologa@gmail.com (P.d.S.d.R.); kelypicoli@gmail.com (K.d.P.S.); 2 Research Group in Biochemistry and Toxicology in Caenorhabditis elegans, Federal University of Pampa, BR 472, km 585, Caixa Postal 118, Uruguaiana CEP 97501-970, Rio Grande do Sul, Brazil; ddani34@hotmail.com (D.A.A.); avilads1@gmail.com (D.S.Á.); 3 Laboratory of Natural Products and Mass Spectrometry, Federal University of Mato Grosso do Sul, Cidade Universitária, Mato Grosso do Sul CEP 79070-900, Brazil; carloscarollo@gmail.com (C.A.C.); denise.brentan@ufms.br (D.B.S.); 4 Polytechnic Institute of Bragança, Agricultural College of Bragança, Campus Santa Apolónia, 5301-855 Bragança, Portugal; leticia@ipb.pt

**Keywords:** fruit native, antioxidant, protects effects, *C. elegans*, nutraceutical food

## Abstract

Fruits are sources of bioactive compounds that are responsible for several biological activities. Therefore, this study aimed to identify the chemical composition of the pulp of the Brazilian Savanna fruit *Dipteryx alata*; evaluate its toxic effects, influence on the life expectancy of the nematode *Caenorhabditis elegans*, and its antioxidant activities in vitro and in vivo; and describe the mechanisms involved. The chemical compounds identified include phenols, terpenes, fatty acid derivatives, vitamins, and a carboxylic acid. The in vitro antioxidant activity was demonstrated by radical scavenging methods. in vivo, the *D. alata* fruit pulp was not toxic and promoted resistance to oxidative stress in nematodes exposed to a chemical oxidizing agent. Furthermore, it promoted an increased life expectancy in wild-type nematodes and increased the expression of superoxide dismutase and the nuclear translocation of DAF-16. These results suggest that the beneficial effects identified are related to these two genes, which are involved in the regulation of metabolic activities, the control of oxidative stress, and the lifespan *of C. elegans*. These beneficial effects, which may be related to its chemical constituents, demonstrate its potential use as a functional and/or nutraceutical food.

## 1. Introduction

Brazil is the country that harbors the world’s greatest plant biodiversity: approximately 20% of the global total. The Cerrado biome, also known as the Brazilian savanna, holds approximately 10,000 of these species, many of them endemic [1]. In recent years, the search for improved quality of life has stimulated the consumption of natural products, which has made Brazilian biodiversity the focus of several studies in the search for plant species with nutritive and functional properties. It is estimated that a typical diet provides more than 25,000 bioactive constituents, which are usually found in vegetables and fruits but also in cereal grains, nuts, and other foods. In this scenario, fruit species play an important role in human nutrition as a source of dietary fibers and essential nutrients. Therefore, nutrition may play an essential role in protecting against damage caused by oxidative stress [2].

Oxidative stress is a result of the excess production or inability to efficiently eliminate reactive oxygen species, which may be localized or systemic, and it causes damage to cellular components such as DNA, proteins, and lipids. This process is described as one of the main triggering and/or aggravating agents of many diseases, such as cancer, atherosclerosis, cardiac diseases, neurodegenerative diseases such as Alzheimer’s, and the aging process [3]. Cellular protection against damage caused by reactive oxygen species is guaranteed through a highly efficient antioxidant defense system, which ensures a balance between pro and antioxidant compounds. These systems are formed by molecular and enzymatic components of endogenous origin, which will differ in concentration and location content, and exogenous natural antioxidants, which are present in foods such as fruits and vegetables.

One rarely studied Brazilian flora species with nutritive and functional potential is *Dipteryx alata* Vogel, which is known as baru or cumbaru and belongs to the family Fabaceae. The fruits and nuts are consumed and used in the production of sweets by the local population. In folk medicine, the bark is used to treat snake bites [4], and the nut oil is used to combat high fever [5], as a menstrual regulator, and in the treatment of rheumatism [6]. Scientific studies have shown that the nut is able to reduce abdominal fat in women [7], scavenge free radicals in vitro, and prevent iron-induced oxidative stress in vivo [8]. However, there are no studies describing the chemical constituents and biological properties of the fruit pulp.

Thus, the objectives of this study were to describe the chemical composition of the fruit pulp of *D. alata* (FPDA); evaluate its toxic effects, ability to increase the life expectancy in the nematode *C. elegans,* and antioxidant activities in vitro and in vivo; and determine the mechanisms involved.

## 2. Materials and Methods

### 2.1. Plant Material and Pulp Preparation

To obtain FPDA, the fruits were collected in the Cerrado, which is located in the municipality of Dourados (21° 59′ 41.8” S and 55° 19′ 24.9” W), state of Mato Grosso do Sul, Brazil, under the permission of the Brazilian National System of Management of Genetic Heritage and Associated Traditional Knowledge (Sistema Nacional de Gestão do Patrimônio Genético e do Conhecimento tradicional associado, SisGen; permit number AA6FADF). The species was identified by a plant taxonomist, and a voucher specimen was deposited in the herbarium (DDMS-UFGD) of the Federal University of Grande Dourados, Dourados (MS), Brazil, under the registration number 5342. The fruits were washed in running water to remove impurities and sanitized by immersion in Sumaveg ^®^ solution (3.3 g/L of water) for 15 min, followed by washing with drinking water. Following sanitization, the pulp was lyophilized and stored at −80 °C. The lyophilized pulp yield was 67%, which was calculated using the following equation:

$$Lyophilized\;pulp\;yield\;\left(\%\right)=\frac{\;weight\;of\;the\;fresh\;pulp\;\left(g\right)\;\times \;100}{weight\;resulting\;from\;the\;lyophilization\;process\;\left(g\right)}$$

### 2.2. Sample Preparation

For the experimental assays, 0.005 g of FPDA was mixed with 5 mL of ultrapure sterile water. The material was mixed and homogenized for 5 min in a vortex mixer. The homogenates were placed in containers away from light and refrigerated at 4 °C for 24 h. The FPDA samples were used to determine the lipophilic antioxidant pigments and ascorbic acid. For the analysis of phenolic compounds and flavonoids as well as the antioxidant activity in vitro, FPDA in liquid medium was centrifuged at 5000 rpm for 10 min, and the supernatant was used.

### 2.3. Chemical Composition

#### 2.3.1. Identification of the Constituents by LC-DAD-MS

The sample (80 mg) was extracted with methanol and deionized water added 0.1% formic acid (7:3, *v*/*v*) (3 mL) for 15 min in the ultranosic bath. The sample was centrifuged and filtered on Millex^®^ (PTFE membrane, 0.22 µm) before the chromatographic system injection. A UFLC Prominence Shimadzu coupled to diode array detector (DAD) and a mass spectrometer (MicrOTOF-Q III, Bruker Daltonics, Billerica, MA, USA) was used in the analyses. The chromatographic column was a Kinetex C18 column (2.6 µm, 150 × 2.1 mm, Phenomenex), applying a flow rate of 0.3 mL/min, oven temperature of 50 °C, and injection volume of 7 µL. Ultrapure water (solvent A) and acetonitrile (solvent B), both added 0.1% formic acid (*v*/*v*), were used as mobile phase with the following gradient elution profile: 0–2 min, 3% B; 2–25 min, 3–25% B; 25–40 min, 25–80% B; and 40–43 min at 80% B. For the MS analyses, nitrogen was used as nebulizer gas at 4 Bar, dry gas at 9 L/min, and collision gas. The sample was analyzed in negative and positive ion modes.

#### 2.3.2. Phenolic Compounds and Flavonoids

For this, the Folin–Ciocalteu colorimetric method was used to determine the total phenolic content present in FPDA. For this assay, 2.5 mL of Folin–Ciocalteu reagent (1:10 *v*/*v*, diluted in distilled water) was added to 0.5 mL of FPDA (at a concentration of 500 μg/mL). This solution was incubated in the dark for 5 min. Subsequently, 2.0 mL of 14% aqueous sodium carbonate (Na_2_CO_3_) was added and incubated at room temperature for 120 min, protected from light. The absorbance was measured at 760 nm using a T70 UV/Vis spectrophotometer (PG Instruments Limited, Leicestershire, UK). A calibration curve with gallic acid (0.0004–0.0217 mg/mL) was used as a standard. The concentration of phenolic compounds present in the pulp is expressed in mg of gallic acid equivalents (GAE)/g of pulp. The assay was performed in triplicate.

The total content of flavonoids in the pulp was determined using an ethanol solution of 2% aluminum chloride hexahydrate (AlCl_3_·6H_2_O), of which 4.5 mL was added to 0.5 mL of pulp (at a concentration of 500 µg/mL), and this solution was kept in the dark for 30 min at room temperature. Subsequently, the absorbance was measured at 415 nm (T70 UV/Vis spectrophotometer, PG Instruments Limited, Leicestershire, UK), and the flavonoid quercetin (0.0004–0.0217 mg/mL) was used in preparing the calibration curve. The total content of flavonoids is expressed in mg of quercetin equivalents (QE)/g of pulp. The assay was performed in triplicate.

#### 2.3.3. Lipophilic Antioxidants

The lipophilic antioxidants β-carotene, lycopene, and chlorophyll *a* and *b* were determined using dissolved FPDA (150 mg) vigorously stirred in 10 mL of an acetone–hexane mixture (4: 6, *v*/*v*) for 1 min and filtered through Whatman^®^ Grade 4 qualitative filter paper. The absorbance of the filtrate was measured at 453, 505, 645, and 663 nm. The levels of β-carotene, lycopene, and chlorophyll a and b were calculated using the following equations:

β−carotene=0.216×Abs663−1.220×Abs645−0.304×Abs505+0.452×Abs453Lycopene=−0.0458×Abs663+0.204×Abs645+0.304×Abs505−0.0452×Abs453Chlorophyll a=0.9999×Abs663−0.0989×Abs645Chlorophyll b=−0.328×Abs663+1.7×Abs645

The results are expressed in mg/100 g of pulp.

#### 2.3.4. Determination of the Ascorbic Acid Content

For this, 0.5 g of FPDA was vigorously homogenized in 50 mL of oxalic acid. Next, 20 mL of this solution was transferred to a 50 mL volumetric flask, and the volume was completed with oxalic acid. The mixture was filtered using filter paper (Whatman^®^ Grade 4). The filtrate was used to titrate a solution of 2,6-dichlorophenolindophenol (DCPIP) sodium. The titration was terminated when the color of the solution changed into pink without fading for 15 s. Ascorbic acid was used as the standard solution. The experiment was performed in triplicate. The results were calculated based on the equation below and expressed as mg of ascorbic acid/100 g of pulp.

$$\frac{\mathrm{mg}\;\mathrm{Asc}.\;\mathrm{acid}}{100\;g_{\mathrm{sample}}}=\frac{\mathrm{DCPI}P_{\mathrm{sample}}}{\mathrm{DCPI}P_{\mathrm{standard}}}\times \frac{100}{M_{\mathrm{sample}}}\times \frac{\left(M_{\mathrm{solvent}}+M_{\mathrm{sample}}\right)}{m_{\mathrm{extract}}}\times \frac{50\;mL}{10\;mL}\times F\;F=\frac{M_{AA}}{50}\times \frac{1}{25}\times 10$$

where DCPIP_sample_ and DCPIP_standard_ are the volumes used in the titration of the sample and the standard (mL); M_sample_, M_solvent_, and M_extract_ are the masses of the sample, the solvent added for titration of the sample, and the aliquot of the sample (g); F is the amount of ascorbic acid required to reduce the DCPIP (mg); and M_AA_ is the mass of ascorbic acid (mg).

### 2.4. Antioxidant Activity

#### 2.4.1. 2,2-Diphenyl-1-Picrylhydrazyl (DPPH^•^) Free Radical Scavenging

For this experiment, 200 μL of the FPDA (0.1–1000 µg/mL) was mixed with 1800 μL of 0.11 mM DPPH% solution in 70% ethanol. The absorbance was measured at 517 nm. Ascorbic acid and butylated hydroxytoluene (BHT) were used as reference antioxidants. As a control, 200 μL of the solvent (70% ethanol) was incubated with 1800 μL of the DPPH^•^ solution. Three independent experiments were performed in triplicate. The data were expressed as the concentration necessary to inhibit 50% of the free radical (IC_50_). The percentage of inhibition in relation to the control (DPPH solution (0.11 mM) was calculated using the following equation:

$$Capture\;activity\;of\;DPPH^{•}\;\;\left(\%\right)=\left(1-\frac{Abs\;control\;}{Abs\;sample}\right)\times 100$$

#### 2.4.2. Discoloration of the 2,2′-azino-bis-3-ethylbenzothiazoline-6-sulfonic acid (ABTS^•+^) Radical

For this experiment, the ABTS^•+^ was prepared from a mixture of 5 mL of ABTS (7 mM) and 88 μL of potassium persulfate (140 mM), which was incubated for 12–16 h at room temperature in the dark. After this period, the ABTS^•+^ solution was diluted in absolute ethanol until an absorbance of 0.70 ± 0.05 was obtained at 734 nm. Then, 20 μL of the FPDA (0.1–1500 µg/mL) was added to 1980 μL of the ABTS^•+^. The final concentrations of the extract ranged from 0.1 to 200 μg/mL. The mixture was incubated at room temperature in the dark for 6 min. The absorbance was evaluated at 734 nm. Ascorbic acid and BHT were used as positive controls. As a control, 20 μL of the solvent (80% ethanol) was incubated with 1980 μL of ABTS^•+^. Three independent experiments were performed in triplicate. The percentage of ABTS^•+^ inhibition was calculated according to the following equation:

$$Inhibition\;of\;ABTS^{•+}\;\left(\%\right)=\left(\frac{Abs\;control-Abs\;sample}{Abs\;control}\right)\times 100$$

### 2.5. In Vivo Assays

#### 2.5.1. Caenorhabditis Elegans Strains, Maintenance, Synchronization, and Experimental Controls

The in vivo assays were performed with N2 wild-type and transgenic CF1553, CL2166, and TJ356 *C. elegans* nematodes obtained from the *Caenorhabditis* Genetics Center (CGC), Minnesota, USA. The nematodes were kept in incubators at 20 °C, grown in Petri dishes containing nematode growth medium (NGM), and fed *Escherichia coli* OP50 bacteria. The bacteria used in the assays were inactivated with the antibiotic kanamycin (10 mM).

The nematode culture was synchronized with 2% sodium hypochlorite and 5 M sodium hydroxide. The eggs resistant to alkaline lysis were collected and transferred to Petri dishes containing NGM and *E. coli* with different concentrations of FPDA or solvent (water) used in pulp preparation.

In the assays with *C. elegans,* the observed biological effects were evidenced by comparing the treatment with FPDA (at a concentration of 500 μg/mL and 1000 μg/mL) with the solvent used as a negative control. A positive control (pharmacological control) was not used in vivo, and the activities of FPDA or oxidizing agent juglone were compared to those on the untreated nematode *C. elegans*, which is an experimental protocol widely used in the literature [9,10,11].

#### 2.5.2. Toxicity

To evaluate the toxicity assay, on average 10 age-synchronized N2 nematodes were transferred to 96-well microplates containing M9 culture medium (100 μL) and FPDA (100 μL) at different concentrations (10 to 1000 μg/mL). Next, the nematodes were incubated at 20 °C for 24 and 48 h. As a negative control, the nematodes were incubated with only M9 culture medium (200 μL). After the incubation period, the nematode viability was assessed by sensitivity to touch using a platinum wire. The results of three independent assays performed in triplicate were averaged.

#### 2.5.3. Number of Progeny

To evaluate the total number of progeny, five synchronized N2 nematodes treated with FPDA (500 and 1000 μg/mL) or with the solvent (water) used as a negative control were individually transferred to new dishes containing *E. coli* with or without FPDA. The nematodes were transferred daily to new dishes for five days (egg-laying period). The number of progeny was evaluated in each dish after reaching the L3 or L4 larval stages. The average of three independent assays was obtained.

#### 2.5.4. Resistance to Heat Stress

The heat stress resistance assay was performed using synchronized wild-type N2 nematodes treated with FPDA (500 and 1000 μg/mL) or solvent (negative control). Twenty nematodes were transferred to new dishes containing NGM medium, E. coli OP50, and the respective treatments. Heat stress was induced by increasing the culture temperature from 20 to 37 °C over a 6 h period, with an evaluation performed each hour. After the incubation period, the nematodes were kept at 20 °C for at least 16 h. This procedure allows the viable nematodes to recover. The results of three independent assays performed in duplicate were averaged.

#### 2.5.5. Resistance to Oxidative Stress

The oxidative stress assay was performed by exposing the nematodes to the oxidizing agent juglone (5-hydroxy-1,4-naphthoquinone). Synchronized N2 nematodes treated with FPDA (500 and 1000 μg/mL) or solvent (negative control) in two different manners were used: in the first, the nematodes were treated with FPDA from eggs until the L4 phase, and treatment was interrupted at the time of stress induction; in the second, the initial treatment was the same, but it was not interrupted at the time of stress induction. From these, 10 nematodes were transferred to 96-well microplates containing M9 culture medium (200 μL) and juglone (50 μL), corresponding to the condition with interrupted treatment, and 10 nematodes were transferred to 96-well microplates containing M9 culture medium (100 μL), FPDA (100 μL), and juglone (50 μL), corresponding to the condition with continued treatment. All nematodes were incubated at 20 °C for 6 h. The evaluations were performed every hour of the experiment. Nematodes incubated in only M9 culture medium (250 μL) and with juglone (200 μL M9 + 50 μL juglone) were used as controls. The viability of the nematodes was evaluated by sensitivity to touch using a platinum wire. The results from three independent assays performed in triplicate were averaged.

#### 2.5.6. Lifespan Assay

Synchronized N2 nematodes treated with FPDA (500 and 1000 μg/mL) or only the solvent (negative control) were used. On the first day of the adult stage, 20 nematodes per group were transferred to new Petri dishes with E. coli with or without FPDA, which was considered experimental day 1. Over the first six days, the nematodes were transferred daily to new dishes of the respective treatment, during which time the animals were in the reproduction phase. Starting on the seventh day, the nematodes were transferred every 2–3 days. The survival evaluation consisted of classifying the nematodes as dead/alive daily until all of the nematodes died. The nematodes were considered dead when they did not move with or without stimulation. The nematodes with hatched eggs inside or that disappeared were excluded from analysis. The results from two independent assays performed in triplicate were averaged.

#### 2.5.7. Expression of SOD-3 and GST-4 Proteins

The transgenic strains CF1553 (muls84[pAD76(sod-3::GFP)]) and CL2166 (dvls19[pAF15(gst-4::GFP::NLS)]) were used to quantify the expression of the antioxidant enzymes superoxide dismutase (SOD-3) and glutathione transferase (GST-4). The nematodes were synchronized and treated with FPDA (500 and 1000 μg/mL) or only with the solvent (negative control). After 48 h, the treated nematodes were transferred to microscope slides (containing 1 mM levamisole as anesthetic), and the images were analyzed using an epifluorescence microscope (Nikon Eclipse 50i) connected to a digital camera (Samsung ST 64). Images of five nematodes from each group were taken, and the relative fluorescence of the entire body was determined using ImageJ software. Three independent assays were performed in triplicate.

#### 2.5.8. Expression of the Transcription Factor DAF-16

The transgenic strain TJ356 (zIs356[pGP30(DAF-16::GFP)+pRF4(rol-6)]) was used to evaluate the nuclear translocation of transcription factor DAF-16. The nematodes were synchronized and treated with FPDA (500 and 1000 μg/mL) or only with the solvent (negative control). After 48 h, the treated nematodes were transferred to microscope slides (containing 1 mM levamisole as anesthetic), and the images were analyzed using an epifluorescence microscope (Nikon Eclipse 50i) connected to a digital camera (Samsung ST 64). Images of five nematodes from each group were taken, and the relative fluorescence of the entire body was determined using ImageJ software. Three independent assays were performed in triplicate.

### 2.6. Statistical Analysis

The data are expressed as the mean ± standard error of the mean (SEM). The 50% inhibitory concentrations (IC_50_) with 95% confidence limits were determined by nonlinear regression. Student’s *t*-test was used to compare the control and treatment, and univariate analysis of variance (ANOVA) followed by Dunnett’s post-test were used to compare two or more groups. The survival curve was constructed by log-rank test analysis (Mantel–Cox). GraphPad Prism 5 software (San Diego, CA, USA) was used to compile and analyze all of the data. The results were considered significant when *p* < 0.05.

## 3. Results

### 3.1. Identification of the Constituents by LC-DAD-MS

**Figure 1:**
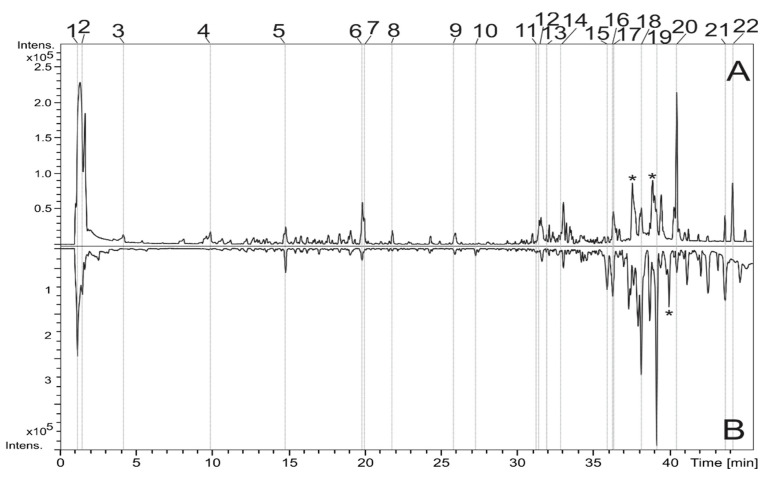
Total ion chromatograms from fruit pulp of *D. alata* (FPDA) obtained in the negative (**A**) and positive ion mode (**B**) (* peaks are not from the sample).

The extract from fruit pulp of *D. alata* (FPDA) was obtained with methanol and deionized water with 0.1% formic acid added, and it was analyzed by LC-DAD-MS. From DAD analyses, the UV spectral data were obtained for each constituent, but only chromatograms obtained from MS (positive and negative ion modes) were illustrated because the sensitivity of MS is higher than UV, and some constituents did not revealed UV absorption. Twenty-two compounds were detected and identified from *D. alata* (Figure 1; Table 1), including flavonoids, phenols, terpenes derivatives, and fatty acids.

The peaks 1 and 2 revealed intense ions at *m/z* 341.1103 and 191.0206 [M-H]^−^, which were compatible to the molecular formula C_12_H_22_O_11_ and C_6_H_8_O_7_ and they were putatively identified as di-*O*-hexoside and citric acid, respectively. These compounds have been described from *D. alata* [12].

Compound 3 showed a band at the wavelength 280 nm in the UV spectrum, suggesting a phenolic compound. The ion at *m/z* 315.0735 [M-H]^−^, relative to C_13_H_16_O_9_, produced the fragment ion at *m/z* 153 by the losses of a hexoside molecule. The spectral data were compatible to *O*-hexosyl protocatechuic acid, which was isolated from the Fabaceae family [13]. In addition, the peak 9 revealed a co-elution of two substances, and one of them exhibited UV spectrum (λ_max_ ≈ 285 and 335 nm) characteristics of flavone. From the ion *m/z* 285.0413 [M-H] (C_15_H_9_O_6_^−^), the observed fragment ions were compatible to flavone luteolin [14], which has been described from the genus *Dipteryx* [15].

Compound 10 revealed an intense ion at *m/z* 315.0859 [M+H]^+^, which is relative to the molecular formula C_17_H_14_O_6_. Its fragment ion *m/z* 300 is yielded from methyl radical loss, confirming the methoxyl group. Thus, compound 10 was putatively identified as di-*O*-methoxy dihydroxy isoflavone, which is a compound described from *D. alata* [5].

### 3.2. Chemical Composition

The concentrations of the evaluated compounds were phenolic compounds 262.089 ± 0.60 mg GAE/100 g, lycopene 0.033 ± 0.001 µg/g, chlorophyll A 0.082 ± 0.002 µg/g, chlorophyll B 0.090 ± 0.005 µg/g, and ascorbic acid 113.48 ± 15.91 mg/100 g. The presence of flavonoids and β-carotene could not be detected by the methods used.

### 3.3. Antioxidant Activity

#### Neutralization of DPPH^•^ and ABTS^•+^ Radicals

The IC_50_ values of the DPPH and ABTS radicals by FPDA are shown in Table 2. FPDA showed a lower IC_50_ in the ABTS radical discoloration method, indicating that it is approximately 5.5 times more efficient in the scavenging of this radical.

### 3.4. Assays in C. Elegans

#### 3.4.1. Toxicity

**Figure 2:**
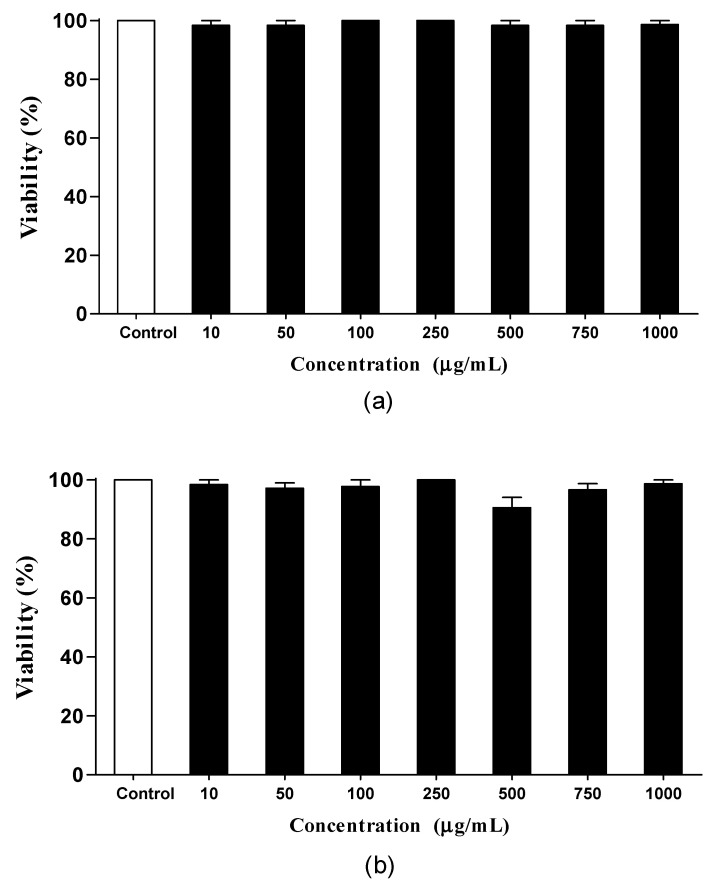
Toxicity of FPDA to *C. elegans* for (**a**) 24 h and (**b**) 48 h. Values are expressed as the mean ± SEM.

The first parameter evaluated was *the* in vivo toxicity. The nematodes were exposed to FPDA during the adult stage (L4) for 24 (Figure 2a) and 48 h (Figure 2b). No toxic effects were observed at the concentrations tested (10 to 1000 μg/mL) in *C. elegans*, which allowed us to proceed safely to the next assessments.

#### 3.4.2. Number of Progeny

**Figure 3:**
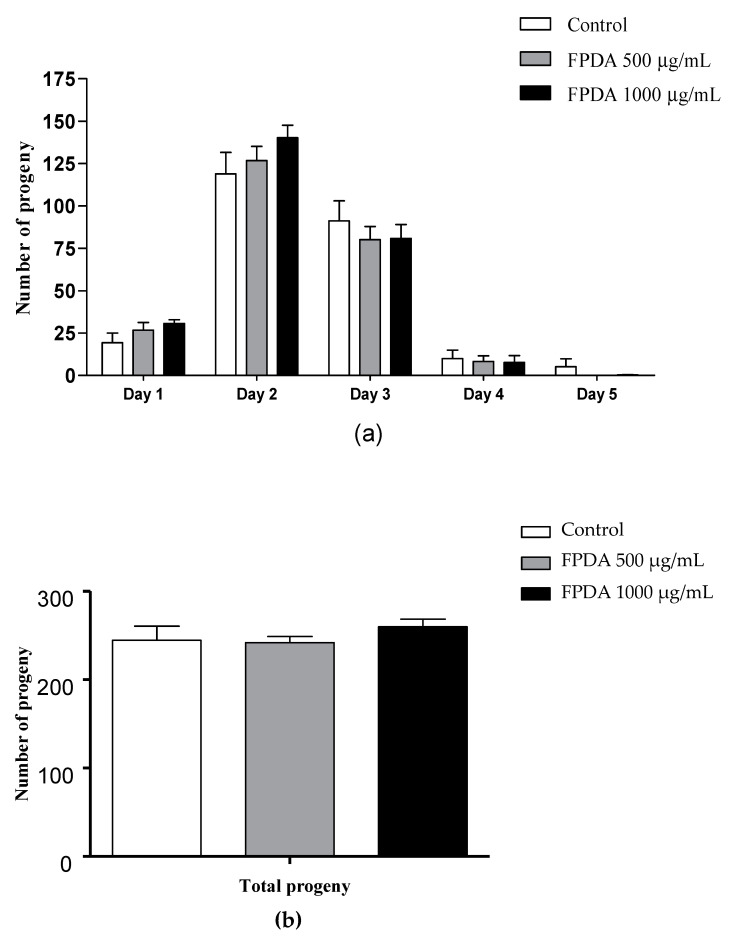
Number of progeny of *C. elegans* treated with FPDA. (**a**) Daily number of progeny and (**b**) total number of progeny after five days. Values are expressed as the mean ± SEM.

To evaluate other toxicity parameters, we analyzed the effects of FPDA on the reproductive capacity. At the concentrations evaluated, no changes were observed in the daily (Figure 3a) or total number (Figure 3b) of descendants of the nematodes treated with FPDA.

#### 3.4.3. Resistance to Heat Stress

**Figure 4:**
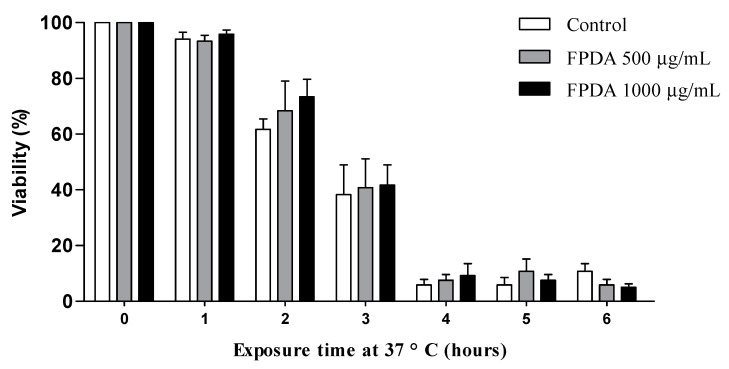
Thermal stress in the in vivo experimental model *C. elegans* subjected to treatment with FPDA. Values are expressed as the mean ± SEM.

The antioxidant properties of FPDA were also evaluated in vivo. For this evaluation, the nematodes treated with FPDA during the egg phase until the L4 larval stage were subjected to heat stress, which is capable of promoting protein damage, at 37 °C for a maximum period of 6 h. In this assay, a protective effect of FPDA on nematode survival could not be observed (Figure 4).

#### 3.4.4. Resistance to Oxidative Stress

**Figure 5:**
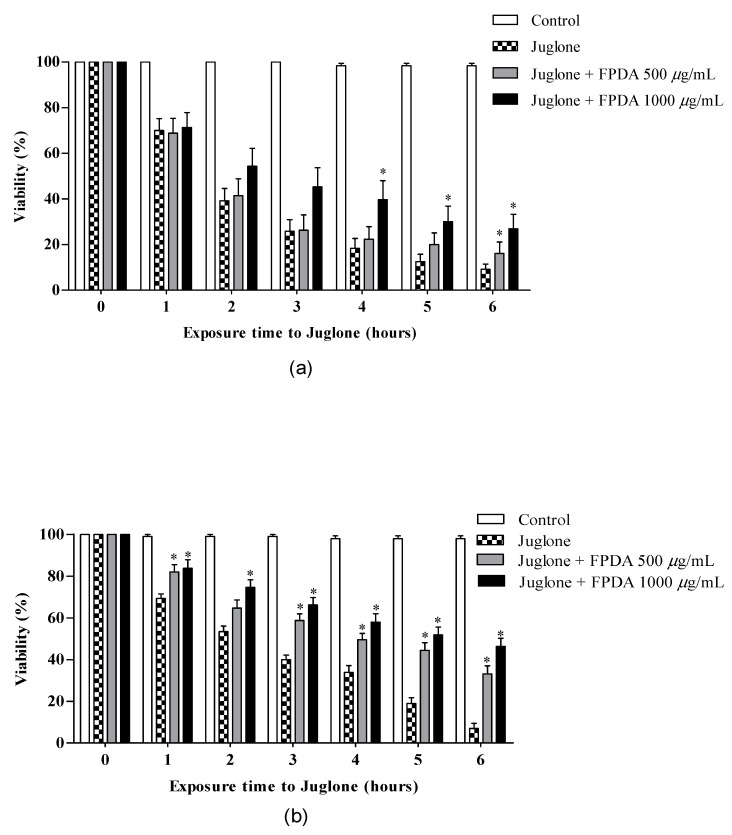
Oxidative stress in the in vivo experimental model C. elegans treated with FPDA from eggs until the L4 phase. (**a**) Treatment with FPDA interrupted at the time of stress induction with juglone; (**b**) Treatment with FPDA not interrupted at the time of stress induction with juglone. * Statistically significant results (*p* < 0.05) when the treated group was compared with the control group. Values are expressed as the mean ± SEM.

In the oxidative stress assay, the nematodes were exposed to juglone, which is a xenobiotic agent that is able to generate reactive oxygen species, which are lethal to nematodes. In the treatment interrupted at the time of stress induction, FPDA at a concentration of 1000 μg/mL increased the nematode resistance against oxidative stress, and it was effective during the periods of 4, 5, and 6 h of evaluation (Figure 5A). When the FPDA treatment was continuous during stress induction with juglone, we observed protection starting from the first hour of evaluation at both concentrations, which extended until the last hour of evaluation, except in the second hour, when there was no significant difference in protection mediated by the pulp at the 500 μg/mL concentration (Figure 5B).

#### 3.4.5. Lifespan

**Figure 6:**
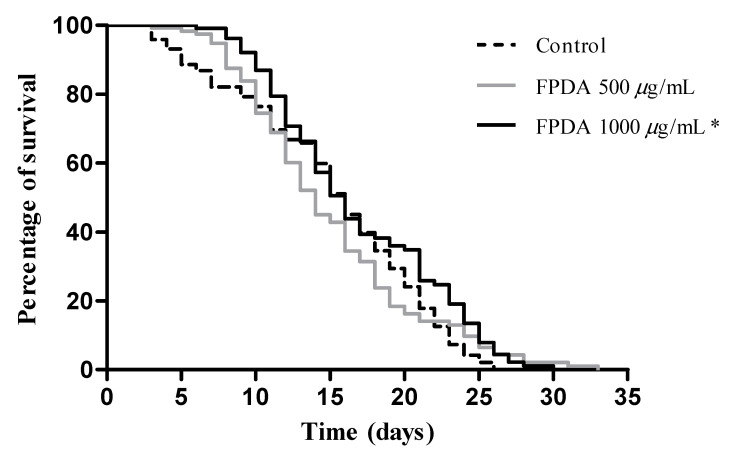
Lifespan assay in the in vivo experimental model of *C. elegans* subjected to treatment with FPDA. * Statistically significant results (*p* < 0.05) when the treated group was compared with the control group.

It has been reported that greater resistance to different stresses may be associated with an increased life expectancy. Therefore, we evaluated the effect of continuous treatment with FPDA on the lifespan of N2 nematodes. We observed that the nematodes treated with FPDA showed a longer lifespan (Figure 6). While the nematodes of the control group lived for a maximum period of 26 days, the nematodes treated with FPDA at a concentration of 500 μg/mL lived for 29.5 days, representing a 13.4% increase in the lifespan. The nematodes treated with a concentration of 1000 μg/mL lived for 29 days, which represented an increase in the lifespan of 11.4%.

#### 3.4.6. Expression of the SOD-3 and GST-4 Proteins

**Figure 7:**
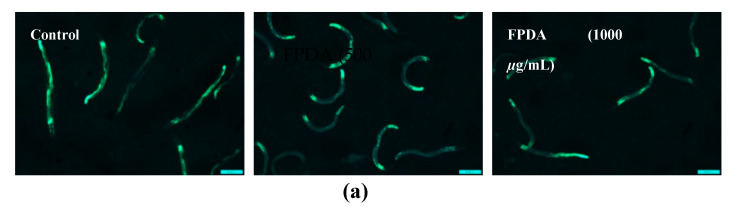

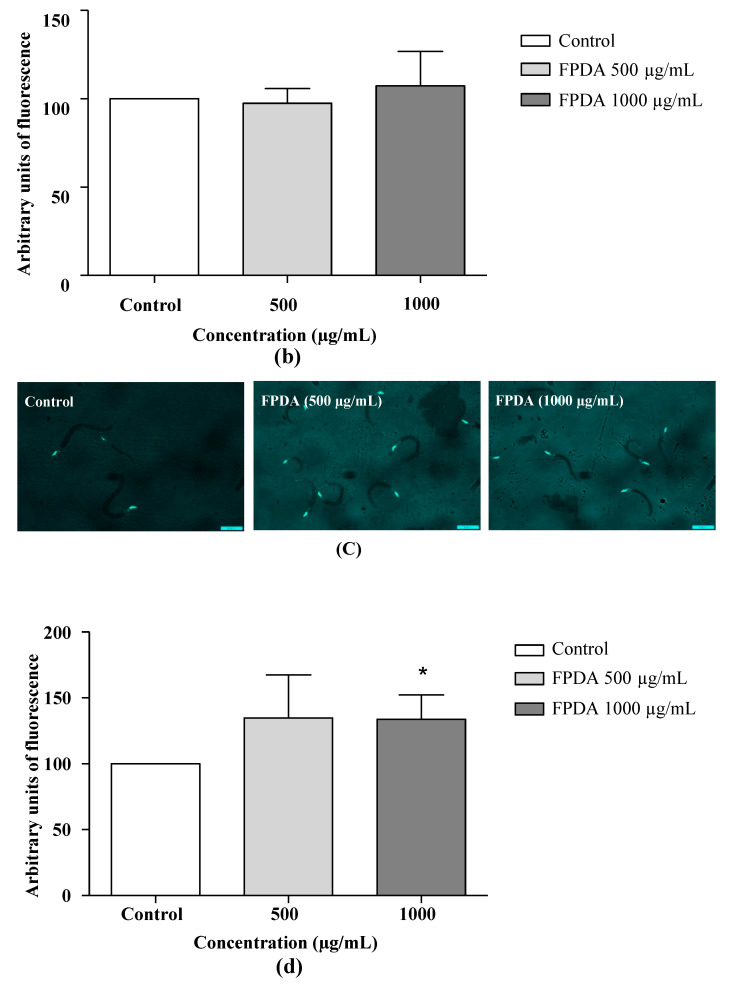
Glutathione transferase (GST-4) and superoxide dismutase (SOD-3) expression in the in vivo experimental model of *C. elegans* subjected to treatment with FPDA. (**a**) Images representative of the expression of GST-4:GFP in treated and untreated nematodes. (**b**) Quantification of GST-4:GFP in treated and untreated nematodes. (**c**) Images representative of the expression of SOD-3:GFP in treated and untreated nematodes; (**d**) Quantification of SOD-3:GFP in treated and untreated nematodes * Statistically significant results (*p* < 0.05) when the treated group was compared with the control group.

The levels of expression of the detoxifying protein GST-4 and antioxidant enzyme SOD-3 were determined using strain CF1553, which expresses fluorescence spots around the head, tail, and vulva, and strain CL2166, which expresses fluorescence spots throughout the body. None of the pulp concentrations affected the expression of GST-4 (Figure 7a,b). Treatment with 1000 μg/mL FPDA resulted in a 33.6% increase in SOD-3 expression compared to the control group (Figure 7c,d), corroborating the previously reported antioxidant data.

#### 3.4.7. Expression of Transcription Factor DAF-16

**Figure 8:**
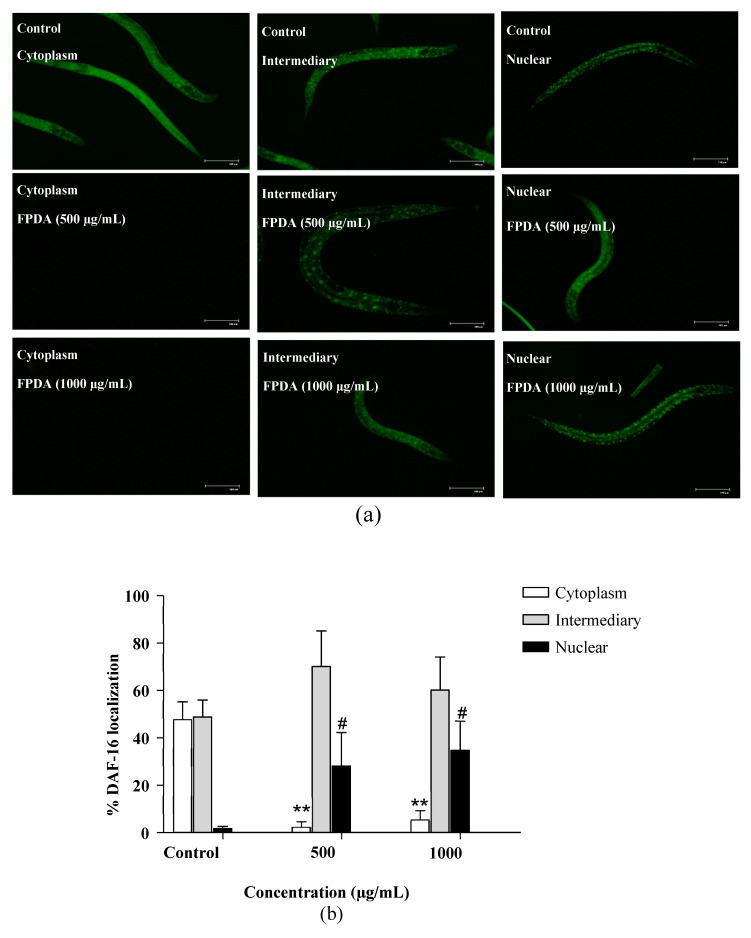
Expression of transcription factor DAF-16 (cytosol, intermediary, and nuclear) in the in vivo experimental model of *C. elegans* subjected to treatment with *D. alata* fruit. (**a**) Representative images of the expression of transcription factor DAF-16:GFP in the cytosol, intermediary, and nucleus of treated and untreated nematodes. (**b**) Quantification of the expression of the transcription factor DAF-16:GFP in the cytoplasm, intermediary, and nucleus of treated and untreated nematodes. ^#^ *p* < 0.05 and ** *p* < 0.01 represents statistically significant results when the treated group was compared with its respective control group.

In the previous experiments, some possible targets involved in the antioxidant potential, resistance to stress, and lifespan regulation were investigated. FPDA stimulated the expression of the antioxidant enzyme SOD-3, which is a gene downstream of DAF-16; thus, the transcription factor DAF-16 was also investigated. In strain TJ356 (Figure 8A), which allows fluorescence visualization through its translocation to the nucleus, treatments with both 500 and 1000 μg/mL FPDA stimulated the nuclear translocation of DAF-16, representing approximately 16- and 21-fold increases compared to the control (Figure 8B).

## 4. Discussion

Aging is a physiological process linked to lifespan, in which a series of undesirable chemical and biochemical events occur, leading to a functional decline of various components of the organism. Several hypotheses have been proposed to explain the mechanisms involved in aging, including the function of free radicals and their oxidizing action on macromolecules such as DNA, proteins, and lipids; the relationship between cellular senescence and deficient energetic metabolism; and the increase in the number of inflammatory cytokines, resulting in diseases associated with age, such as cancer, atherosclerosis, and metabolic diseases. In this sense, alternatives are sought, especially of natural origin, that are capable of preventing or slowing the effects of aging, thus providing better quality of life and a longer lifespan among humans.

The regular consumption of fruits and vegetables is associated with a reduction in the number of cases of chronic diseases. This benefit is generated in large part by the presence of secondary metabolites with biological properties, such as phenolic compounds, carotenoids, sterols, and saponins, among others, providing an equilibrium of the intestinal microbiota and antimicrobial, antioxidant, anti-inflammatory, anticarcinogenic, anti-atherosclerotic, cardioprotective, and neuroprotective action [16].

Thus, the pulp of *D. alata*, a typical fruit of the Brazilian savanna, which is still poorly consumed by the population, was the object of study.

The chemical constituents identified in this study belong to the classes of phenolic acids, flavonoids, terpenes, organic acids, and fatty acid derivatives. Although of the levels of phenolic compounds were identified, these data should be seen as a measure of total antioxidant capacity rather than phenolic content, since that the Folin–Ciocalteu reagent used in this assay is significantly reactive toward other compounds besides phenols as ascorbic acid [17]. In addition, given that the UV-spectrophotometric measures are lower sensitivity than LC-MS [18], the flavonoids were identified only in the most sensitive method. Phenolic compounds are efficient in hydrogen donation, thus neutralizing reactive oxygen species. Among the terpenes, the carotenoid lycopene has the highest antioxidant capacity because it acts by eliminating free radicals such as the superoxide anion and peroxyl radicals [19]. Ascorbic acid can act as a cofactor in several enzymatic reactions, efficiently reducing free radicals derived from cellular respiration; meanwhile, citric acid, found mainly in citric fruits, has chelating and buffering characteristics, thus preventing the browning of food and prolonging its shelf life [20].

Fatty acids are classified according to the number of double bonds in their structure as saturated, unsaturated, or polyunsaturated. Some saturated fatty acids participate in the regulation of cellular metabolism and intracellular signaling [21]. Unsaturated fatty acids, such as omega-3, are involved in the prevention and treatment of several chronic diseases, which may be associated with the integration and modulation of cell membrane signaling proteins [22].

The antioxidant activity of FPDA was demonstrated in vitro through the scavenging of DPPH^•^ and ABTS^•+^ free radicals. These radicals can be neutralized by the donation of electrons or hydrogen atoms. In addition to hydrophilic antioxidant compounds, FPDA has lipophilic compounds such as carotenoids and chlorophylls [23]. The presence of these compounds may explain the higher antioxidant activity in the ABTS^•+^ radical discoloration assay, because this method has selectivity for both water-soluble and fat-soluble antioxidants.

In vivo experimental models are tools that contribute to the understanding of the action of natural products in whole organisms. Among these models, *C. elegans* is one of the alternatives that minimizes the number of mammals used in scientific research. Previous studies have shown that *C. elegans* is an excellent in vivo experimental model to complement in vitro studies and can be used to obtain rapid results in toxicological analyses of pharmacological compounds and the beneficial effects of natural products [24].

Toxicity tests are important because they allow harmful agents in foods and pharmaceuticals to be detected and defined. These studies include fundamentally different approaches, such as in vitro assays in cells and in vivo assays using animals, promoting advances in food safety and drug development, because many drugs already in use are derived from natural products. In *C. elegans,* well-established parameters of toxicity include viability, L4 development (adult stage), and egg laying. Our results show that FPDA did not alter the viability or number of nematode progenies, and no toxic effects were observed on the parameters at the concentrations evaluated. These results enabled the safe analysis of the potential beneficial effects of FPDA in subsequent assays.

The antioxidant activity using *C. elegans* was evaluated by heat and oxidative stress assays. Exposure to high temperature, exceeding the ideal body temperature, is defined as heat shock [25] and is capable of causing some dysfunctions in the body, such as protein denaturation and aggregation as well as the release of Ca^2+^ [26]. Heat stress, as well as other stressors, activates the heat shock response (HSR) and initiates the action of heat shock proteins (Hsps). These proteins are chaperones involved in proteostasis, which act in this process to prevent cellular degeneration and increase heat protection [27].

FPDA was unable to reduce the damage caused by the increase in temperature, although this fruit presents different antioxidant compounds, mainly phenols, which are capable of acting endogenously in the activation of defense systems and the regulation of cell signaling [28]. A similar effect was observed in the study by Bonomo et al. [9], in which it was also not possible to observe an increase in heat tolerance in *C. elegans* through treatment with an aqueous extract of açaí, which is a fruit that is typical of the Amazon region and with a marked presence of phenols.

These results may be associated with the drastic increase in temperature to 37 °C, which is almost double the ideal survival temperature and is described as a lethal dose for most larval stages of *C. elegans*, for up to 3 h. The larval stage is also a variable, and L4 larval stage nematodes are more sensitive to heat stress than those in the L1 phase [19].

Unfavorable changes due to environmental contaminants can lead to cellular oxidative stress, hampering the continuity of metabolic and/or bioenergetic processes. In response to the harmful effects caused by reactive species, cellular defense mechanisms are activated, including the antioxidant system, which includes exogenous compounds such as vitamins, carotenoids, and flavonoids, and endogenous antioxidant enzymes such as SOD, catalase (CAT), and glutathione peroxidase (GPX). These antioxidants act by protecting or repairing macromolecules that are targets of the action of these reactive species in cells [29]. In *C. elegans,* this cellular response also modifies and improves the expression of transcription factors and other molecules, such as heat shock proteins. This action ensures a normal life course for the nematodes until the stress generated by the reactive species exceeds and thus limits this adaptive protection of the biological system. This limitation explains the acute toxicity caused by xenobiotic compounds, such as juglone, and consequently the early death of nematodes.

Thus, the nematodes were next exposed to oxidative stress with juglone (5-hydroxy-1,4-naphthoquinone). This compound is found in plants of the family Juglandaceae and is a secondary metabolite with herbicidal effects that are capable of generating the superoxide anion radical (O_2_^•−^) in the intracellular redox cycle [30]. FPDA was able to protect the nematodes and mitigate the damage caused by reactive oxygen species, regardless of the continuity of treatment with FPDA. It can be postulated that the effect of FPDA on oxidative stress is associated with its direct antioxidant capacity, as shown by the results obtained in the in vitro DPPH^•^ and ABTS^•+^ free radical scavenging assays. In addition, flavonoids, such as the luteolin present in FPDA and luteolin-7-glucoside, have been reported to decrease intracellular ROS production [31], and diterpenes, such as andrographolide lactone, have antioxidant activity [32].

Our results demonstrated that the FPDA evaluated has antioxidant activity in vitro and in vivo. It has been suggested that increasing the resistance to different stresses may be associated with an increased lifespan [33]. In addition, studies such as this one that use *C. elegans* to investigate the effects of plant products on lifespan enable future experiments in higher animals, such as mammals, because they allow observation of their efficacy and mechanisms of action in the production of anti-aging drugs due to the genetic homology of these animal models [18]. From this perspective, the nematodes that were treated daily with FPDA surprisingly showed an increase in lifespan.

This prolongation of the nematode lifespan may be attributed to the presence of the bioactive compounds identified. Several studies have reported that extracts rich in phenolic compounds, such as *Paullinia cupana* (guarana) [10] and green coffee [34], are able to prolong the lifespan of nematodes. Ascorbic acid has also been reported to increase the lifespan in *C. elegans*, *Drosophila melanogaster*, mice, and rats [35].

Genes associated with the lifespan process, such as DAF-16 and SKN-1, homologous to the human genes FOXO and NFR2, respectively, can modulate the lifespan [36]. Flavones such as luteolin have been reported to act on the expression of genes such as Akt/Foxo3a and Nrf2 [37]. DAF-16 and other components are regulated by the insulin/IGF-1 signaling (IIS) pathway. Adverse situations and internal stressors, such as caloric restriction or the interruption of signals transduced to DAF-16, block the IIS pathway and intensify the transcriptional activity of DAF-16, leading to its translocation to the nucleus. This cascade of events has an important action on homeostasis, resistance to different stresses, and an increased lifespan [38]. Thus, we analyzed the nuclear translocation of DAF-16 in nematodes treated with FPDA, and a significant increase in this factor was observed in both treatments, suggesting its role in promoting an increased lifespan.

DAF-16 is considered a master regulator due to its influence on the transcription of several genes [39], such as the antioxidant proteins glutathione-S-transferase and superoxide dismutase. In FPDA, the increasing trend at the highest concentration did not affect the expression of GST-4 is one of the most important enzymes involved in the phase II detoxification of both endogenous oxidative stress and xenobiotic metabolism. GST binds to glutathione and catalyzes the binding of glutathione to the target substrate, aiding its excretion by the cell [40]. However, by evaluating the detoxifying functions of GST-4, it can be suggested that FPDA has no toxic effects on the nematodes. In contrast, the enzyme superoxide dismutase acts by converting the superoxide anion radical produced in the mitochondria into hydrogen peroxide, and the enzyme glutathione-S-transferase acts to detoxify oxidative stress products [41]. Similarly to other natural products derived from Brazilian biodiversity, such as *Eugenia uniflora* L. (pitanga) [11] and *Paullinia cupana* (guarana) [30], FPDA increased the expression of SOD-3, one of the isoforms of manganese superoxide dismutase, indicating one of the possible mechanisms of DAF-16-mediated antioxidant activity. Both genes are related to metabolic activity regulation, oxidative stress control, and the lifespan in *C. elegans.*

## 5. Conclusions

The results of this study indicate that the fruit pulp of *D. alata* has different bioactive compounds with antioxidant activity, which was confirmed both in vitro and *in vivo,* and it is capable of increasing the lifespan expectancy of *C. elegans* nematodes via SOD-3 and DAF-16, demonstrating its potential for use as a functional and/or nutraceutical food.

## Figures and Tables

**Table 1:** Identification of the constituents from fruit pulp of *D. alata* (FPDA) by LC-DAD-MS/MS. DAD: diode array detector.

Peak	RT (min)	Compound	UV (nm)	MF	Negative Mode (*m/z*)		Positive Mode (*m/z)*	
					MS [M-H]^-^	MS/MS	MS [M+H]^+^	MS/MS
1	1.2	di-*O*-hexoside	-	C_12_H_22_O_11_	341.1103	179	365.1052 ^a^	-
2	1.5	Citric acid	-	C_6_H_8_O_7_	191.0206	-	193.0331	
3	4.1	***O*-hexosyl protocatechuic acid**	288	C_13_H_16_O_9_	315.0735	153		
4	9.9	**NI**	-	C_17_H_22_O_12_	417.1073	152	441.1012 ^a^	-
5	14.8	**Vicenin 2**	270, 335	C_27_H_30_O_15_	593.1544	503, 473, 383, 353, 325, 297	595.1694	541, 481, 457, 439, 409, 391, 379, 355, 337, 325, 295
6	19.9	**NI**	282	C_18_H_22_O_10_	397.1154	249, 189	399.1301	223
7	20.0	**NI**	286, 330	C_9_H_16_O_4_	187.0985	-	189.1127	171
8	21.8	**Coumaric acid derivative**	290, 318	C_24_H_28_O_12_	507.1526	231, 203, 163	509.1626	-
9	25.9	**NI**	285, 335	C_16_H_30_O_6_	317.1985	263, 237, 219, 171		
		**Luteolin**		C_15_H_10_O_6_	285.0413	257, 239, 199, 175, 151	287.0564	153
10	27.3	**di-*O*-methoxy dihydroxy isoflavone**	280	C_17_H_14_O_6_	313.0730	-	315.0859	300, 243, 167
11	31.5	**Fatty acid derivative**	-	C_18_H_34_O_5_	329.2349	229, 211, 183, 171	-	-
12	31.7	**Diterpene**	285	C_20_H_28_O_5_	347.1880	285, 259	349.2005	285, 239, 187, 161
13	32.1	**NI**	285	C_21_H_30_O_6_	377.1983	333, 301, 263	379.2130	361, 283, 213, 161
14	33.1	**NI**	285	C_22_H_32_O_6_	391.2133	287, 191	393.2267	315, 297, 269, 213, 199, 171, 161
15	35.9	**NI**	-	C_21_H_30_O_4_	-	-	347.2220	287, 269, 251, 187, 163
16	36.3	**NI**	-	C_19_H_28_O_3_	303.1966	252, 205		
17	36.3	**NI**	-	C_21_H_30_O_4_	-	-	347.2219	287, 269, 243, 187, 163
18	38.2	**NI**	-	C_45_H_94_N_6_O_17_	-	-	991.6737	
19	39.2	**NI**	-	C_22_H_34_O_4_	-	-	361.2372	301, 283, 245, 199, 171
20	40.5	**Fatty acid derivative**	-	C_20_H_30_O_2_	301.2186	-	-	-
21	43.6	**Hexadecanoic acid**	-	C_16_H_32_O_2_	255.2341			
22	44.2	**Octadecenoic acid**	-	C_18_H_34_O_2_	281.2491			

RT: retention time; MF: molecular formula; NI: non-identified; ^a^ [M + Na]^+^.

**Table 2:** Antioxidant activity of *D. alata* fruit pulp. ABTS: 2,2′-azino-bis-3-ethylbenzothiazoline-6-sulfonic acid, BHT: butylated hydroxytoluene, DPPH: 2,2-diphenyl-1-picrylhydrazyl, FPDA: fruit pulp of *D. alata.*

Samples	DPPH^•^	ABTS^•+^
	IC_50_ (µg/mL)	IC_50_ (µg/mL)
Ascorbic acid	2.65 ± 0.20	1.43 ± 0.09
BHT	14.58 ± 2.15	10.15 ± 0.94
FPDA	2306.33 ± 101.83	416.0 ± 28.00

Values are expressed as the mean ± SEM.
